# Health decision-making capacity and modern contraceptive utilization among sexually active women: Evidence from the 2014–2015 Chad Demographic and Health Survey

**DOI:** 10.1186/s40834-022-00188-7

**Published:** 2022-10-24

**Authors:** Kenneth Setorwu Adde, Edward Kwabena Ameyaw, Barbara Elorm Mottey, Mawulorm Akpeke, Roberta Mensima Amoah, Nafisatu Sulemana, Kwamena Sekyi Dickson

**Affiliations:** 1 Department of Population and Health, College of Humanities and Legal Studies, University of Cape Coast, Cape Coast, Ghana; 2 Institute of Policy Studies and School of Graduate Studies, Lingnan University, Tuen Mun, New Territories Hong Kong; 3 L & E Research Consult Ltd, Upper West Region, Wa, Ghana; 4 Institute of Health Research, University of Health and Allied Sciences, Hohoe, Ghana; 5 Department of Environmental Sciences, University of Massachusetts, Amherst, USA; 6 Maternal and Child Health Unit, University Health Services, University for Development Studies, Tamale, Ghana; 7 DKT International, Accra, Ghana

**Keywords:** Women, Chad, Modern contraception, Reproductive health, Demographic and Health Survey

## Abstract

**Background:**

Globally, there has been an increase in the percentage of women in their reproductive ages who need modern contraceptives for family planning. However, in Chad, use of modern contraceptive is still low (with prevalence of 7.7%) and this may be attributable to the annual increase in growth rate by 3.5%. Social, cultural, and religious norms have been identified to influence the decision-making abilities of women in sub-Saharan Africa concerning the use of modern contraceptives. The main aim of the study is to assess the association between the health decision-making capacities of women in Chad and the use of modern contraceptives.

**Methods:**

The 2014–2015 Chad Demographic and Health Survey data involving women aged 15–49 were used for this study. A total of 4,113 women who were in sexual union with information on decision making, contraceptive use and other sociodemographic factors like age, education level, employment status, place of residence, wealth index, marital status, age at first sex, and parity were included in the study. Descriptive analysis and logistic regression were performed using STATA version 13.

**Results:**

The prevalence of modern contraceptive use was 5.7%. Women who take health decisions with someone are more likely to use modern contraceptives than those who do not (aOR = 2.71; 95% CI = 1.41, 5.21). Education, ability to refuse sex and employment status were found to be associated with the use of modern contraceptives. Whereas those who reside in rural settings are less likely to use modern contraceptives, those who have at least primary education are more likely to use modern contraceptives. Neither age, marital status, nor first age at sex was found to be associated with the use of modern contraceptives.

**Conclusion:**

Education of Chad women in reproductive age on the importance of the use of contraceptives will go a long way to foster the use of these. This is because the study has shown that when women make decisions with others, they are more likely to opt for the use of modern contraceptives and so a well-informed society will most likely have increased prevalence of modern contraceptive use.

**Plain language summary:**

The use of modern contraceptives remains a pragmatic and cost-effective public health intervention for reducing maternal mortality, averting unintended pregnancy and controlling of rapid population growth, especially in developing countries. Although there has been an increase in the utilization of modern contraceptives globally, it is still low in Chad with a prevalence rate of 7.7%. This study assessed the association between the health decision-making capacities of women in Chad and the use of modern contraceptives. We used data from the 2014 − 2015 Chad Demographic and Health Survey. Our study involved 4,113 women who were in sexual union and with complete data on all variables of interest. We found the prevalence of modern contraceptive utilization at 5.7%. Level of education of women, women who can refuse sex and employment status were found to be significantly associated with the use of modern contraceptives. Whereas those who reside in rural settings are less likely to use modern contraceptives, those who have at least primary education are more likely to use modern contraceptives. Our study contributes to the efforts being made to increase the utilisation of modern contraceptives. There is a need to step up contraceptive education and improve adherence among Chad women in their reproductive years. In the development of interventions aiming at promoting contraceptive use, significant others such as partners and persons who make health decisions with or on behalf of women must be targeted as well.

## Background

Women in their reproductive age with the need for family planning satisfied by modern contraceptive method has seen a steady increase globally, from 73.6% in the year 2000 to 76.8% in the year 2020 [1, 2]. Some reasons ascribed to this mild change include limited access to services, as well as cultural and religious factors [3]). However, these barriers are being addressed in some regions, and this accounts for an increase in demand for modern methods of contraception [2].

According to the World Health Organization, the proportion of women with needs for modern methods of contraception has been stagnant at 77% from 2015 to 2020 [2]. Globally, the number of women using modern contraceptive methods has increased from 663 million in 2000 to 851 million in 2020 [2]. In 2030, it is projected that an additional 70 million women will be using a modern contraceptive method [2]. In low to middle-income countries, the 214 million women who wanted to avoid pregnancy were not using any method of contraception as of 2020 [2]. Low levels of contraceptive use have mortality and clinical implications [3]. However, about 51 million in their reproductive age have an unmet need for modern contraception [1]. Maternal death and new born mortality could be reduced from 308,000 to 84,000 and 2.7 million to 538,000 respectively, if women with intentions to avoid pregnancy were provided with modern contraceptives [4].

Low prevalence in the use of modern contraceptives has been linked to negative events such as maternal mortality and unsafe abortion in Africa [5–7]. Women with low fertility intentions in sub-Saharan Africa, record the lowest prevalence rate of modern contraceptive use [8].

The use of modern contraceptive remains a pragmatic and cost-effective public health intervention for reducing maternal mortality, averting unintended pregnancy and controlling rapid population growth especially in developing countries [3, 9]. Beson, Appiah & Adomah-Afari, [3] highlight that knowledge and awareness per se do not result in the utilization of modern contraceptives [3]. Cultural and religious myths and misconceptions tend to undermine the use of modern contraception [10–12]. Ensuring access and utilization of contraceptives has benefits extending beyond just the health of the population [3]. Amongst these include sustainable population growth, economic development and women empowerment [2, 3].

Nonetheless. predominantly in SSA, women do not have the decision-making capacity to make decisions pertaining to their health [13]. Although this has proven to be an efficient driver for improved reproductive health outcomes for women [14, 15]. To improve efforts on contraceptive usage in Africa, people are encouraged to make positive reproductive health decisions to prevent unintended pregnancy and other sexually transmitted infections since these steps would lead to the reduction of maternal mortality and early childbirth amongst women [14].

At an estimated population growth of 3.5% per year, Chad’s population growth is considered to be growing at a relatively fast pace [16]. This trend in growth may be ascribed to the country’s high fertility and low use of contraceptives [16]. In Africa, Chad had been found to have the lowest prevalence of modern contraceptive use in sub-Saharan Africa despite its recorded growth from 5.7% to 2015 to 7.7% in 2019 [8, 17]. UN Women [18] data also showed that a lot need to be done in Chad to achieve gender equality with about 6 out of 10 women aged 20–24 years married before age 18 and about 165 of women in their reproductive age reporting being victims to physical and/or sexual violence by a current or former intimate partner in the year 2018. Studies indicate that social and religious norms have undermined women’s rights and self-determination in Chad [19-21] which affects their health decision-making capacity negatively. This situation makes it challenging for women in their reproductive age in Chad to be of independent mind when making decisions about the use of modern contraception. Though the use of contraception in many parts of the world had been found to yield immense benefits such as low levels of maternal mortality and morbidity, and to a larger extent influence economic growth and development [2, 3]. It has become necessary to investigate the health decision-making capacity and modern contraceptive utilisation among sexually active women in Chad. Findings from this study will provide stakeholders and decision-makers with evidence that will guide policymaking to improve access and utilisation of modern contraceptives in Chad.

## Materials and methods

### Data source

The study used data from the current Demographic and Health Survey (DHS) conducted in Chad 2104 − 2015. The 2014–2015 Chad Demographic and Health Survey (CDHS) aimed at providing current estimates of basic demographic and health indicators. It captured information on health decision making, fertility, awareness, and utilization of family planning methods, unintended pregnancy, contraceptive use, skilled birth attendance, and other essential maternal and child health indicators [22].

The survey targeted women aged 15–49 years. The study used DHS data to provide holistic and in-depth evidence of the relationship between health decision-making and the use of modern contraceptives in Chad. DHS is a nationwide survey collected every five-year period across low- and middle-income countries. A stratified dual-stage sampling approach was employed. Selection of clusters (i.e., enumeration areas [EAs]) was the first step in the sampling process, followed by systematic household sampling within the selected EAs. For the purpose of this study, only women (15–49 years) in sexual unions (marriage and cohabitation) who had complete cases on all the variables of interest were used. The total sample for the study was 4,113.

### Study variables

#### Dependent variable

The dependent variable in this study was “contraceptive use” which was derived from the ‘current contraceptive method’. The responses were coded 0 = “No method”, 1 = “folkloric method”, 2 = “traditional method,” and 3 = “modern method”. The existing DHS variable excluded women who were pregnant and those who had never had sex. The modern methods included female sterilization, intrauterine contraceptive device (IUD), contraceptive injection, contraceptive implants (Norplant), contraceptive pill, condoms, emergency contraception, standard day method (SDM), vaginal methods (foam, jelly, suppository), lactational amenorrhea method (LAM), country-specific modern methods, and respondent-mentioned other modern contraceptive methods (e.g., cervical cap, contraceptive sponge). Periodic abstinence (rhythm, calendar method), withdrawal (coitus interruptus), and country-specific traditional methods of proven effectiveness were considered as traditional methods while locally described methods and spiritual methods (e.g., herbs, amulets, gris-gris) of unproven effectiveness were the folkloric methods. To obtain a binary outcome, all respondents who said they used no method, folkloric, traditional, were put in one category and were given the code “0 = No” whereas those who were using the modern method were also put into one category and given the code “1 = Yes.”

#### Explanatory variables

Health decision-making capacity was the main explanatory variable. For health decision-making capacity, women were asked who usually decides on respondent’s health care. The responses were respondent alone, respondent and husband/partner, husband/partner alone, someone else, and others. This was recorded to respondent alone = 1, respondent and someone (respondent and husband/partner, someone else, and others) = 2, and partner alone = 3. Similarly, some covariates were included based on theoretical relevance and conclusions drawn about their association with modern contraceptive utilisation [13, 14, 23]. These variables are age, place of residence, wealth quintile, employment status, educational level, marital status, age at first sex, and parity.

### Analytical technique

We analysed the data using STATA version 13. We started with descriptive computation of modern contraception utilization with respect to health decision-making capacity and the covariates. We presented these as frequencies and percentages (Table 1). We conducted Chi-square tests to explore the level of significance between health decision-making capacity, covariates, and modern contraceptive utilization at a 5% margin of error (Table 2). In the next step, we employed binary logistic regression analysis in determining the influence of health decision-making capacity on modern contraceptive utilization among women in their reproductive ages as shown in the first model (Model I in Table 3). We presented the results of this model as crude odds ratios (cOR) with their corresponding 95% confidence intervals. We further explored the effect of the covariates to ascertain the net effect of health decision-making capacity on modern contraceptive utilization in the second model (Model III in Table 3) where adjusted odds ratios (aOR) were reported. Normative categories were chosen as reference groups for the independent variables. Sample weight was applied whilst computing the frequencies and percentages so that we could obtain results that are representative at the national and domain levels. We used STATA’s survey command (SVY) in the regression models to cater for the complex sampling procedure of the survey. We assessed multicollinearity among our co-variates with Variance Inflation Factor (VIF) and realized that no multicollinearity existed with a mean VIF = 3.7.

**Table 1:** Background characteristics of respondents and proportion using modern contraceptive

Variable	Frequency (n = 4113)	Percentage (%)
**Health decision maker**		
Respondent alone	355	8.6
Respondent and someone	727	17.7
Partner alone	3031	73.7
**Age**		
15–19	480	11.7
20–24	750	18.2
25–29	876	21.3
30–34	695	16.9
35–39	558	13.6
40–44	432	10.5
45–49	323	7.9
**Level of education**		
No education	2858	69.5
Primary	847	20.6
Secondary or higher	408	9.9
**Place of residence**		
Urban	831	20.2
Rural	3282	79.8
**Marital status**		
Married	3741	91.0
Cohabiting	372	9.0
**Occupation**		
Not working	1919	46.7
Working	2194	53.4
**Wealth status**		
Poorest	685	16.7
Poorer	903	21.9
Middle	898	21.8
Richer	889	21.6
Richest	738	17.9
**Age at first sex**		
Less than 16 years	2362	57.4
16–19 years	1387	33.7
20+	365	8.9
**Parity**		
Zero birth	252	6.1
One birth	471	11.4
Two births	448	10.9
Three births	501	12.2
Four births or more	2440	59.3
**Can refuse sex**		
No	2303	56.0
Yes	1810	44.0

**Table 2:** Modern contraceptive prevalence by socio-demographic characteristics

Socio-demographic characteristics	Use of modern contraceptive		***X***^***2***^**;** ***p-value***
	**No** **n (%)**	**Yes** **n (%)**	
**Health decision maker**			X^2^ = 35.16; p = 0.000
Respondent alone	354 (96.5)	13 (3.5)	
Respondent and someone	618 (92.0)	54 (8.0)	
Partner alone	2978 (96.9)	96 (3.1)	
**Age**			X^2^ = 20.57; p = 0.002
15–19	478 (99.0)	5 (1.0)	
20–24	698 (96.1)	28 (3.9)	
25–29	821 (95.5)	39 (4.5)	
30–34	674 (95.3)	33 (5.9)	
35–39	538 (94.1)	34 (5.9)	
40–44	421 (96.3)	16 (3.7)	
45–49	320 (97.6)	8 (2.4)	
**Level of education**			X^2^ = 114.19; p = 0.000
No education	3039 (97.7)	71 (2.3)	
Primary	634 (92.7)	50 (7.3)	
Secondary or higher	277 (86.8)	42 (13.2)	
**Place of residence**			X^2^ = 61.67; p = 0.000
Urban	802 (91.5)	75 (8.6)	
Rural	3148 (97.3)	88 (2.7)	
**Marital status**			X^2^ = 7.60; p = 0.006
Married	3.686 (96.3)	143 (3.7)	
Cohabiting	264 (96.0)	163 (4.0)	
**Occupation**			X^2^ = 32.87; p = 0.000
Not working	2139 (97.7)	51 (2.3)	
Working	1811 (94.2)	112 (5.8)	
**Wealth status**			X^2^ = 72.35; p = 0.000
Poorest	643 (97.0)	20 (3.0)	
Poorer	822 (97.4)	22 (2.6)	
Middle	900 (97.2)	26 (2.8)	
Richer	948 (97.2)	27 (2.8)	
Richest	637 (90.4)	68 (9.7)	
**Age at first sex**			X^2^ = 6.78; p = 0.034
Less than 16 years	2212 (96.7)	76 (3.3)	
16–19 years	1381 (95.0)	73 (5.0)	
20+	357 (96.2)	14 (3.8)	
**Parity**			X^2^ = 13.5; p = 0.009
Zero birth	267 (99.3)	2 (0.7)	
One birth	440 (97.1)	13 (2.9)	
Two births	437 (96.7)	15 (3.3)	
Three births	478 (96.6)	17 (3.4)	
Four births or more	2328 (95.3)	116 (4.8)	
**Can refuse sex**			X^2^ = 33.33; p = 0.000
No	2374 (97.5)	61 (2.5)	
Yes	1576 (93.9)	102 (6.1)	

**Table 3:** Multivariate logistic regression results on the predictors of modern contraception utilisation among women in Chad

Variables	Model 1	Model 2	Model 3
**Health decision maker**			
Respondent alone	Ref		Ref
Respondent and someone	2.38**[1.28–4.42]		2.71**[1.41–5.21]
Partner alone	0.88[0.49–1.58]		1.49[0.79–2.78]
**Age**			
15–19		Ref	Ref
20–24		2.33[0.82–6.66]	2.52[0.88–7.19]
25–29		2.14[0.69–6.64]	2.14[0.69–6.65]
30–34		2.08[0.64–6.72]	2.15[0.66–6.98]
35–39		2.76[0.84–9.09]	2.89[0.88–9.55]
40–44		1.75[0.49–6.12]	1.77[0.50–6.22]
45–49		1.02[0.26–3.93]	1.06[0.27–4.12]
**Level of education**			
No education		Ref	Ref
Primary		2.45***[1.64–3.66]	2.34***[1.56–3.50]
Secondary or higher		4.28***[2.60–7.03]	4.02***[2.44–6.6]
**Place of residence**			
Urban		Ref	Ref
Rural		0.47**[0.27–0.81]	0.47**[0.27–0.82]
**Marital status**			
Married		Ref	Ref
Cohabiting		1.52[0.90–2.56]	1.61[0.95–2.71]
**Occupation**			
Not working		Ref	Ref
Working		2.37***[1.64–3.43]	2.24***[1.54–3.28]
**Wealth status**			
Poorest		Ref	Ref
Poorer		0.92[0.49–1.74]	0.89[0.48–1.69]
Middle		1.12[0.60–2.06]	1.05[0.57–1.95]
Richer		1.09[0.59–2.01]	1.07[0.58–1.96]
Richest		1.58[0.81–3.08]	1.57[0.81–3.06]
**Age at first sex**			
Less than 16 years		Ref	Ref
16–19 years		1.39[0.97–1.99]	1.41[0.99–2.02]
20+		1.05[0.55–2.01]	1.07[0.56–2.05]
**Parity**			
Zero birth		Ref	Ref
One birth		3.05[0.66–14.16]	3.04[0.66–14.13]
Two births		2.96[0.62–13.98]	2.93[0.62–13.86]
Three births		3.35[0.69–16.28]	3.33[0.69–16.11]
Four births or more		6.79**[1.43–32.38]	6.7*[1.42–31.80]
**Can refuse sex**			
No		Ref	Ref
Yes		1.62**[1.15–2.29]	1.61**[1.14–2.27]

Computed from 2014–2015 Chad Demographic and Health Survey *Ref* reference category

*p < 0.05; **p < 0.01; ***p < 0.001

## Results

### Socio-demographics and prevalence of modern contraceptive use 

**Figure 1:**
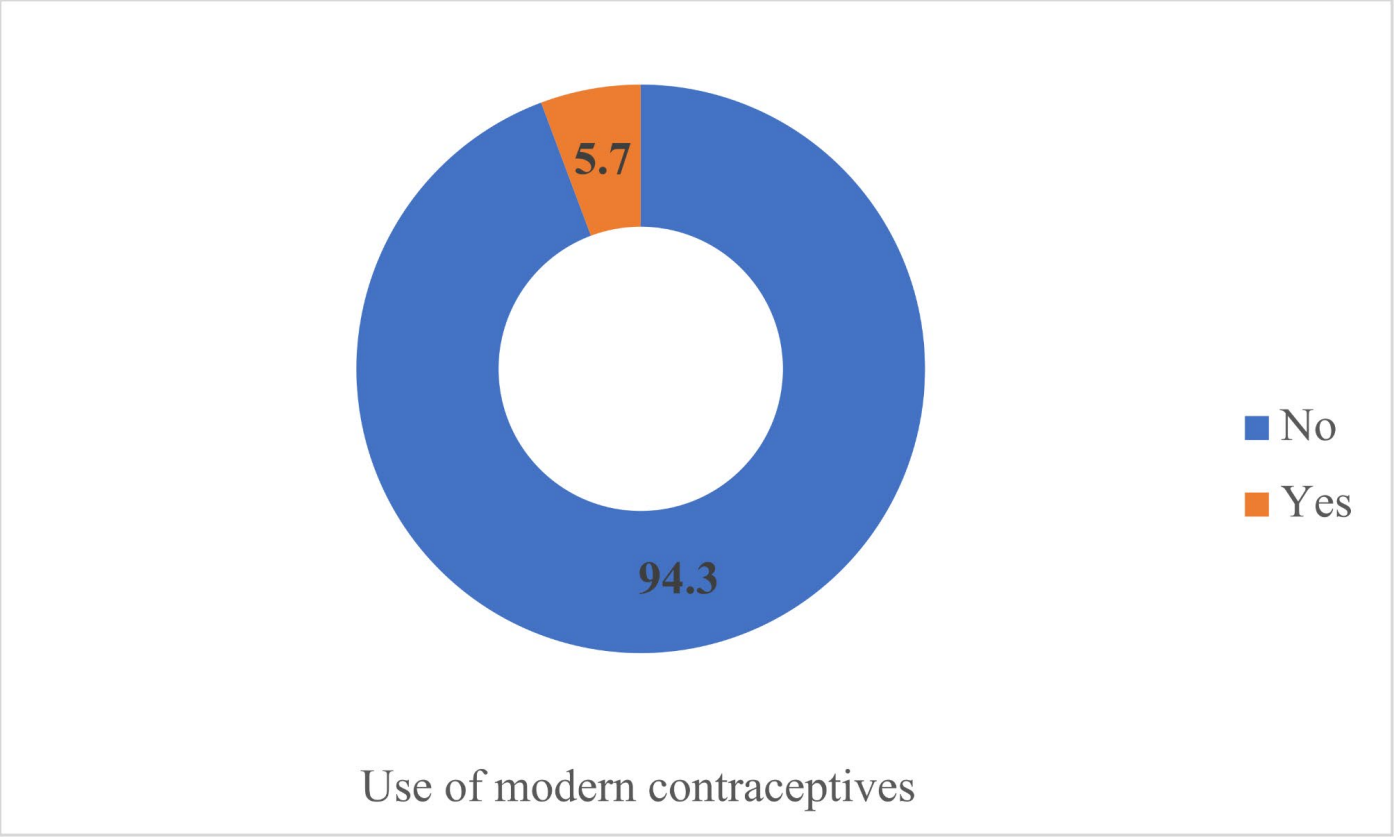
Use of modern contraceptives

Among the respondents who participated in this study, 91% of them are married and about three-quarters of them (73.3%) have partners that are sole decision-makers regarding health issues (Table 1). Most of the respondents (79.8%) reside in rural settings with 69.5% having no education and about half (56.4%) between the ages of 20 and 34 (Table 1). A higher proportion of the respondents (56%) are not able to refuse sex when demanded. The prevalence of modern contraceptive use is 5.7% [CI = 5.46–5.91] (see Fig. 1).

### Association between use of modern contraceptive and the predictor variables

As shown in Table 3, in both the adjusted and the unadjusted models, respondents who take health decisions with someone are more than two times (aOR = 2.71; 95% CI = 1.41, 5.21 and OR = 2.38; 95% CI = 1.28, 4.42 respectively) likely to decide on using contraceptives than respondents who decide alone. It has also been observed that having at least a primary education positively affect the likelihood of using modern contraceptives; primary education (aOR = 2.34; 95% CI = 1.56, 3.50); secondary or higher education (aOR = 4.02; 95% CI = 2.44, 6.60) (see Table 3). Likewise, people who reside in rural areas are 53% less likely to patronize modern contraceptives (aOR = 0.47; 95% CI = 0.27, 0.82) than their counterparts who live in urban areas (see Table 3). Furthermore, women who are employed have higher odds of using contraceptives than those who are unemployed (aOR = 2.24; 95% CI = 1.54, 3.28). Along with that, women who have given birth at least four (4) times are 61% more likely to use modern contraceptives (aOR = 2.71; 95% CI = 1.41, 5.21) than those with no birth experience. It was observed that women who can refuse sex have higher odds of using modern contraceptives (aOR = 1.61; 95% CI = 1.14, 2.27) relative to those who are unable to refuse sex (see Table 3).

## Discussion

This study was essential since the ability of sexually active women to make significant decisions on their health including choices of modern contraceptive use (i.e., condom use) can lead to good reproductive health [24]. We observed that the prevalence of modern contraceptive use in Chad among women in sexual union was 5.7%. Generally, about three-quarters (73.3%) of respondents who were married (91%) had partners as the sole decision-makers regarding their health issues, a finding similar to that of a study conducted in Ghana where only a quarter of women in the study took healthcare decisions single-handedly [25]. However, in a multi-country assessment, it was revealed that about 68.66% of respondents across the 32 nations studied could make decisions on their reproductive health [14]. The discrepancy might be attributable to the diverse research populations and the number of nations investigated.

Furthermore, parties to decision-making regarding the health of women play an important role in the usage of contraceptives. It was found that respondents who took health decisions with someone were more than two times likely to decide on using contraceptives than respondents alone. Similar result was seen in Burkina Faso [26] and Mozambique [27] as spousal decision with women had a positive influence on the utilization of contraception. In terms of decisions taken solely by partners, women were 18% less likely to report an intention to use contraceptives [27]. Again, it was revealed in Pakistan that women whose partners were sole decision-makers were less likely to use contraception. This shows that a woman’s inability to discuss and make decisions on health, especially on family planning issues can negatively affect the use of modern contraception.

Education has been recognised as a strong determining factor of contemporary contraception use. It exposes women to factual information as well as convinces their partners of the need for contraception [28]. This is relevant as we also observed that having at least a primary education induces higher odds of using modern contraceptives. Although the study establish education to be significantly associated with contraption use, a study conducted to measure the trend in the use of contraception in 27 countries in sub-Saharan Africa reported that an increase in the proportion of the study participants with secondary education did not affect the use of contraception [29]. In agreement with our finding, a high level of education has been found to increase the likelihood of using modern ways of delaying birth in women living in Uganda [30]. A plausible explanation to this is that as the level of education of women increases, women are more empowered to take charge of their health decision-making capacity. Since education empowers women to have autonomy over their reproductive rights [23].

With regards to the place of residence, we found that urban women were more likely to use modern contraceptives as compared to their counterparts in rural areas. a possible explanation is that women in urban areas may have better access to information, and are more likely to be interested in education, hence, the use of modern contraceptives to delay childbirth. Other reasons may be poor transportation access, long distances to access health facilities and shortage of contraceptives in the rural areas as compared to in the urban areas [31].This corroborates the findings of Apanga et al., [32] which they ascribed to the fact that there is a high prevalence of late marriage in urban areas as compared to rural areas [33]. Hence, there is a possibility that women in urban areas are likely to use modern contraceptives to avoid unwanted pregnancies.

Consistent with prior studies in Ghana, [3, 34] women who are working had a higher likelihood of utilizing contraception methods than those who are unemployed. The reason for this is because, compared to their non-working peers, the working class may be willing to do everything to maintain their employment and have more time for their occupations instead of having children, especially given their capacity to acquire contraceptives in comparison to their non-working peers. Also, working women are expected to have the financial backing to be able to make health decisions concerning their reproductive health. It is, therefore, no surprise that we found women within the richest wealth quintile to have the highest likelihood of utilizing modern contraceptives.

Finally, women who have given birth to at least four (4) children are more likely to use contraceptives than those with no birth experience. A plausible explanation is that multiparous women may not want more children hence resort to the use of modern contraceptives to either delay the next pregnancy or stop childbirth. This finding is similar to previous reports from Ethiopia and Tanzania which reported that as the number of living children increases, so does the usage of modern contraceptives [35, 36].

## Strength and limitations

We used a large dataset comprising 4,113 women aged between 15–49 which makes our results compelling. Findings from this study are also based on rigorous logistic regression. Despite these strengths, the study had some notable shortcomings. To begin with, the study’s cross-sectional methodology limits causal inferences between respondents’ individual factors and modern contraceptive use. Second, because most questions were answered using the self-reporting approach, there is a risk of social desirability and memory bias in the results. Furthermore, because this study only included women, the conclusions do not incorporate the perspectives of spouses. Finally, we believed that variables like cultural norms and health-care provider attitudes would be relevant to investigate in the context of this study, however, such variables were not included in the DHS dataset.

## Conclusion

The study revealed that modern contraceptive utilization is very low among sexually active women in Chad. We conclude that health decision-makers, education, occupation status [working], higher parity and women’s ability to refuse sex have positive association with modern contraceptive utilization among sexually active women in Chad. There is a need to step up modern contraceptive education and improve adherence among women in their reproductive years. In the development of interventions aiming at promoting modern contraceptive use, broader contextual elements must be prioritized. For instance, significant others such as partners and persons who make health decisions with or on behalf of women need to be targeted.

## Data Availability

Data used for the study is freely available to the public via https://dhsprogram.com/data/available-datasets.cfm.

## Abbreviations

**AIC**: Akaike Information Criterion.
**aOR**: Adjusted Odds Ratio.
**BIC**: Bayesian Information Criterion.
**CDHS**: Chad Demographic and Health Survey.
**cOR**: Crude Odds Ratio.
**DHS**: Demographic and Health Survey.
**EAs**: Enumeration Areas.
**ICC**: Intra-Cluster Correlation.
**IUD**: Intrauterine Device.
**LAM**: Lactational amenorrhea method.
**LMICs**: Lower-and middle-income countries.
**LR**: Likelihood Ratio.
**MLRM**: Multilevel Logistic Regression Model.
**PSU**: Primary Sampling Unit.
**SDM**: Standard Day Method.
**SRHR**: Sexual and Reproductive Health Right.
**SSA**: Sub-Saharan Africa.
**VIF**: Variance Inflation Factor.
**WHO**: World Health Organisation.

## References

[1] Kantorová V, Wheldon MC, Ueffing P, Dasgupta AN (2020). Estimating progress towards meeting women’s contraceptive needs in 185 countries: A Bayesian hierarchical modelling study. PLoS Med.

[2] World Health Organization. WHO recommendations on self-care interventions: self-administration of injectable contraception (No. WHO/SRH/20.9):. World Health Organization; 2020.

[3] Benson P, Appiah R, Adomah-Afari A. Modern contraceptive use among reproductive-aged women in Ghana: prevalence, predictors, and policy implications. BMC women’s health. 2018 Dec;18(1):1–8. 10.1186/s12905-018-0649-2 PMC6156857 30253759

[4] Darroch JE, Sully E, Biddlecom A (2017). Adding it up: investing in contraception and maternal and newborn health, 2017—supplementary tables.

[5] Apanga PA, Adam MA. Factors influencing the uptake of family planning services in the Talensi District, Ghana. Pan African Medical Journal. 2015 Mar 6;20(1). 10.11604/pamj.2015.20.10.5301 PMC4430143 25995807

[6] Eliason S, Awoonor-Williams JK, Eliason C, Novignon J, Nonvignon J, Aikins M (2014). Determinants of modern family planning use among women of reproductive age in the Nkwanta district of Ghana: a case–control study. Reproductive health.

[7] Orach CG, Otim G, Aporomon JF, Amone R, Okello SA, Odongkara B, Komakech H (2015). Perceptions, attitude and use of family planning services in post conflict Gulu district, northern Uganda. Confl health.

[8] Ahinkorah BO, Budu E, Aboagye RG, Agbaglo E, Arthur-Holmes F, Adu C, Archer aG, Aderoju YbG, Seidu A (2021). Factors associated with modern contraceptive use among women with no fertility intention in sub-Saharan Africa: evidence from cross-sectional surveys of 29 countries. Contracept Reprod Med.

[9] Sserwanja Q, Musaba MW, Mukunya D (2021). Prevalence and factors associated with modern contraceptives utilization among female adolescents in Uganda. BMC Womens Health.

[10] Gueye A, Speizer IS, Corroon M, Okigbo CC. Belief in family planning myths at the individual and community levels and modern contraceptive use in urban Africa. International perspectives on sexual and reproductive health. 2015 Dec;41(4):191. 10.1363/4119115 PMC4858446 26871727

[11] Hindin MJ, McGough LJ, Adanu RM. Misperceptions, misinformation and myths about modern contraceptive use in Ghana. Journal of Family Planning and Reproductive Health Care. 2014 Jan 1;40(1):30 – 5. 10.1136/jfprhc-2012-100464 23771916

[12] Wulifan JK, Mazalale J, Kambala C, Angko W, Asante J, Kpinpuo S, Kalolo A. Prevalence and determinants of unmet need for family planning among married women in Ghana-a multinomial logistic regression analysis of the GDHS, 2014. Contraception and reproductive medicine. 2019 Dec;4(1):1–4. 10.1186/s40834-018-0083-8 PMC6352348 30723547

[13] Achana FS, Bawah AA, Jackson EF, Welaga P, Awine T, Asuo-Mante E (2015). Spatial and socio-demographic determinants of contraceptive use in the Upper East region of Ghana. Reproductive health.

[14] Ahinkorah BO, Hagan JE, Seidu AA, Sambah F, Adoboi F, Schack T, Budu E (2020). Female adolescents’ reproductive health decision-making capacity and contraceptive use in sub-Saharan Africa: What does the future hold?. PLoS ONE.

[15] Seidu AA, Ahinkorah BO, Ameyaw EK, Hubert A, Agbemavi W, Armah-Ansah EK, Budu E, Sambah F, Tackie V. What has women’s reproductive health decision-making capacity and other factors got to do with pregnancy termination in sub-Saharan Africa? evidence from 27 cross-sectional surveys. PLoS One. 2020 Jul 23;15(7):e0235329. 10.1371/journal.pone.0235329 PMC7377410 32702035

[16] Yaya AM, Caroline G, Abderahim MN, Solene BH, Doungous DM, Marret H (2020). Use of Female Contraception, Mixed and Multicentric Study in Chad. Am J Public Health.

[17] Yaya S, Uthman OA, Ekholuenetale M, Bishwajit G (2018). Women empowerment as an enabling factor of contraceptive use in sub-Saharan Africa: a multilevel analysis of cross-sectional surveys of 32 countries. Reproductive health.

[18] UN Women Data Hub [Internet]. Country Fact Sheet | Unwomen.org. 2013 [cited 2022 Sep 6]. Available from: https://data.unwomen.org/country/chad ‌

[19] Amzat J. The question of autonomy in maternal health in Africa: a rights-based consideration. Journal of bioethical inquiry. 2015 Jun;12(2):283–93. 10.1007/s11673-015-9607-y 25652571

[20] Hindin MJ, Muntifering CJ. Women’s autonomy and timing of most recent sexual intercourse in Sub-Saharan Africa: a multi-country analysis. Journal of sex research. 2011 Nov 1;48(6):511-9. 10.1080/00224499.2011.554918 21318922

[21] Singh K, Bloom S, Brodish P. Gender equality as a means to improve maternal and child health in Africa. Health care for women international. 2015 Jan 2;36(1):57–69. 10.1080/07399332.2013.824971 PMC4333143 24028632

[22] Institut National de la Statistique, des Études. Économiques et Démographiques (INSEED), Ministère de la Santé Publique (MSP) et ICF International. Enquête Démographique et de Santé et à Indicateurs Multiples (EDS-MICS 2014–2015). Rockville, Maryland, USA: INSEED, MSP et ICF International. 2014–2015.

[23] Mandiwa C, Namondwe B, Makwinja A (2018). Factors associated with contraceptive use among young women in Malawi: analysis of the 2015-16 Malawi demographic and health survey data. Contracept Reproductive Med.

[24] Seidu AA, Ahinkorah BO, Agbemavi W, Amu H, Bonsu F (2021). Reproductive health decision-making capacity and pregnancy termination among Ghanaian women: Analysis of the 2014 Ghana demographic and health survey. J Public Health.

[25] Budu E, Seidu AA, Armah-Ansah EK, Sambah F, Baatiema L, Ahinkorah BO (2020). Women’s autonomy in healthcare decision-making and healthcare seeking behaviour for childhood illness in Ghana: Analysis of data from the 2014 Ghana Demographic and Health Survey. PLoS ONE.

[26] Klomegah R. Spousal communication, power, and contraceptive use in Burkina Faso, West Africa. Marriage & Family Review. 2006 Dec 7;40(2–3):89–105.

[27] Mboane R, Bhatta MP (2015). Influence of a husband’s healthcare decision making role on a woman’s intention to use contraceptives among Mozambican women. Reproductive health.

[28] Asekun-Olarinmoye EO, Adebimpe WO, Bamidele JO, Odu OO, Asekun-Olarinmoye IO, Ojofeitimi EO (2013). Barriers to use of modern contraceptives among women in an inner city area of Osogbo metropolis, Osun state, Nigeria. Int J women’s health.

[29] Emina JB, Chirwa T, Kandala NB. Trend in the use of modern contraception in sub-Saharan Africa: does women’s education matter?. Contraception. 2014 Aug 1;90(2):154 – 61. 10.1016/j.contraception.2014.02.001 24835827

[30] Asiimwe JB, Ndugga P, Mushomi J, Ntozi JP (2014). Factors associated with modern contraceptive use among young and older women in Uganda; a comparative analysis. BMC Public Health.

[31] Bekele D, Surur F, Nigatu B, Teklu A, Getinet T, Kassa M, Gebremedhin M, Gebremichael B, Abesha Y (2021). Contraceptive prevalence rate and associated factors among reproductive age women in four emerging regions of Ethiopia: a mixed method study. Contracept Reproductive Med.

[32] Apanga PA, Kumbeni MT, Ayamga EA (2020). Prevalence and factors associated with modern contraceptive use among women of reproductive age in 20 African countries: a large population-based study. BMJ Open.

[33] Pradhan R, Wynter K, Fisher J (2015). Factors associated with pregnancy among adolescents in low-income and lower middle-income countries: a systematic review. J Epidemiol Community Health.

[34] Nyarko SH. Prevalence and correlates of contraceptive use among female adolescents in Ghana. BMC women’s health. 2015 Dec;15(1):1–6. 10.1186/s12905-015-0221-2 PMC4545366 26286609

[35] Mohammed A, Woldeyohannes D, Feleke A, Megabiaw B (2014). Determinants of modern contraceptive utilization among married women of reproductive age group in North Shoa Zone, Amhara Region, Ethiopia. Reproductive health.

[36] Lwelamira J, Mnyamagola G, Msaki MM. Knowledge. Attitude and Practice (KAP) towards modern contraceptives among married women of reproductive age in Mpwapwa District, Central Tanzania. Current Research Journal of Social Sciences. 2012 May 10;4(3):235 – 45.

